# Supplementary material for: Complexome profiling of the Chlamydomonas psb28 mutant reveals TEF5 as an early PSII assembly factor
Source: Plant Cell. 2025 Mar 17;37(6):koaf055. doi: 10.1093/plcell/koaf055 (PMC12164590; doi:10.1093/plcell/koaf055)
Supplement: koaf055_Supplementary_Data [file koaf055_supplementary_data.zip › psb28_interactive.zip!/plots/intensity/qualityPlots/peptides/_Cre04.g213985.t1.1.html]

### Cre04.g213985.t1.1

Trivial name:   
SeqCoverage [median]: 21.10  
Mass: 24.82 kDa  
  
 

| Sequence | MissedCleavage | Mass [Da] | wtIntensity | muIntensity | Score |
| --- | --- | --- | --- | --- | --- |
| AAYDTILR | 0 | 921.49 | 758.93 | 778.26 | 67.03 |
| AHGMGMDFGEGGPVR | 0 | 1516.66 | 0.00 | 438.19 | 54.90 |
| DAVGINPFAIR | 0 | 1171.63 | 1950.51 | 0.00 | 100.02 |
| TAYEVLGLDTAASR | 0 | 1465.74 | 386.26 | 1290.25 | 128.01 |
|  |  |  | 3095.7 | 2506.7 |  |
